# Association between visual hallucinations and α‐synuclein oligomers in patients with dementia with Lewy bodies

**DOI:** 10.1002/alz.70904

**Published:** 2025-11-14

**Authors:** Hiroaki Sekiya, Lukas Franke, Daisuke Ono, Michael DeTure, Owen A. Ross, Gregory S. Day, Christian Lachner, Neill R Graff‐Radford, Pamela J. McLean, Tanis J. Ferman, Dennis W. Dickson

**Affiliations:** ^1^ Department of Neuroscience Mayo Clinic Jacksonville Florida USA; ^2^ Department of Neurology Mayo Clinic Jacksonville Florida USA; ^3^ Department of Psychiatry and Psychology Mayo Clinic Jacksonville Florida USA

**Keywords:** α‐synuclein, dementia with Lewy bodies, oligomers, visual hallucinations

## Abstract

**INTRODUCTION:**

Visual hallucinations (VHs) represent one of the core clinical features of dementia with Lewy bodies (DLB); however, their underlying pathology remains unclear.

**METHODS:**

We employed proximity ligation assay (PLA) and phosphorylated α‐synuclein (αSYN) immunohistochemistry to compare αSYN oligomers and Lewy‐related pathology across brain regions along the ventral visual pathway in patients with and without VHs (five patients each).

**RESULTS:**

Greater αSYN oligomer burden in the parahippocampal cortex was observed in patients with VHs compared to those without (*p* = 0.041), whereas the burden of Lewy‐related pathology was similar between groups.

**DISCUSSION:**

Our findings suggest that αSYN oligomers, rather than conventional Lewy‐related pathology, may be more closely associated with VHs in DLB. This provides novel evidence linking αSYN oligomers to a core clinical feature in DLB and suggests potential therapeutic targets for managing VH in these patients.

**Highlights:**

- Visualization of αSYN oligomers by PLA and quantitative neuropathologic analysis.
- Abundant αSYN oligomers in parahippocampal cortex of DLB with VHs.
- First human brain study linking αSYN oligomers to VHs.
- Potential oligomer‐targeted therapy for managing VH in DLB.

## 1 INTRODUCTION

Dementia with Lewy bodies (DLB) is the second most common form of dementia following Alzheimer's disease dementia. Clinical features of this condition include dementia, cognitive fluctuations, rapid eye movement sleep behavior disorder, parkinsonism, and visual hallucinations (VHs).^1^ Approximately two‐thirds of patients with DLB develop VHs, which is a higher percentage than seen in other neurodegenerative conditions, including Parkinson's disease (PD) and Alzheimer's disease.^2^, ^3^ VHs have been associated with worse performance on attention‐executive and visual processing tasks.^4^, ^5^ Furthermore, VHs in DLB have been associated with functional impairment, greater caregiver distress, diminished quality of life, and increased hospitalization.^6^ Despite their clinical importance, the underlying pathology of VHs remains unclear.

The pathologic hallmark of DLB is Lewy‐related pathology (Lewy bodies and Lewy neurites), which is mainly composed of α‐synuclein (αSYN).^1^, ^7^, ^8^ These intracytoplasmic inclusions can be clearly visualized using αSYN immunohistochemistry.^9^, ^10^ Previous studies examined the relationship between Lewy‐related pathology and VHs and suggested dysfunction in regions associated with visual processing.^11^, ^12^, ^13^, ^14^ For instance, higher Lewy body counts have been observed in the parahippocampal gyrus and inferior temporal lobe in DLB patients with VHs,^11^ and more severe αSYN deposition has been observed in the deeper layers of the superior colliculus.^13^ Still, other studies have found no significant differences in the severity of Lewy‐related pathology between DLB patients with and without VHs,^12^ suggesting other contributing factors such as cholinergic depletion^15^ or another form of αSYN aggregates. While Lewy‐related pathology is composed of αSYN fibrils and considered to be late‐stage aggregates of αSYN, there is increasing evidence of greater toxicity in earlier‐stage aggregates composed of αSYN oligomers than Lewy‐related pathology.^16^, ^17^, ^18^, ^19^, ^20^ Since detection of αSYN oligomers requires specialized staining techniques, the relationship between clinical symptoms and αSYN oligomers in human α‐synucleinopathies remains poorly understood. We have visualized αSYN oligomers in brain sections using αSYN proximity ligation assay (PLA) staining.^21^, ^22^ Using this specialized staining, we observed greater hippocampal αSYN oligomer accumulation in patients with PD with cognitive impairment compared to those without.^23^ In DLB, patients with rapid cognitive decline showed more αSYN oligomers in the hippocampal CA1 region compared to those with slow decline.^24^

In this study, we sought to better understand the underlying pathology of VHs in DLB. Given the relationship between ventral visual pathway and VHs shown by functional and structural neuroimaging studies,^25^, ^26^, ^27^, ^28^ we hypothesized that αSYN oligomer accumulation along the ventral visual pathway might play an important role in the development of VHs in patients with DLB. To examine this association, we compared the burden of Lewy‐related and αSYN oligomer pathologies in autopsied brains from patients with DLB who experienced fully formed VHs versus those who did not.

## 2 MATERIALS AND METHODS

### 2.1 Study subjects

The study included 10 prospectively and longitudinally followed patients, as part of the Mayo Clinic Alzheimer's Disease Research Center in Jacksonville, Florida, with autopsy‐confirmed Lewy body disease who met the clinical criteria for DLB.^1^ For comparison purposes, the sample was based on the most recent autopsies and included five individuals who experienced VHs during life and five individuals with no history of VHs. All patients completed annual clinical evaluations within 3 years of death, including interviews with a reliable collateral source, comprehensive neurologic examination, and neuropsychological assessment.^29^ To assess cognitive impairment in both groups, we calculated the annualized change in Mini‐Mental State Examination (MMSE) scores using linear regression across all available assessments.^24^, ^30^

### 2.2 Neuropathologic assessment

All cases underwent standardized neuropathologic evaluation by a board‐certified neuropathologist (D.W.D.), which included both macroscopic observation and microscopic assessments.^31^, ^32^ The microscopic assessments included hematoxylin and eosin (H&E) staining, thioflavin S fluorescent microscopy, and immunohistochemistry with an antibody against αSYN (NACP, rabbit polyclonal, 1:3000, with formic acid pretreatment).^33^ Based on thioflavin S fluorescent microscopy, Braak neurofibrillary tangle stage^34^ and Thal amyloid phase^35^ were determined. The pathological diagnosis of Lewy body disease was established based on the presence of Lewy bodies in vulnerable brain regions on H&E‐stained slides and NACP‐stained slides.^36^, ^37^ We assessed transactive response DNA binding protein of 43 kDa (TDP‐43) pathology using a phosphorylated TDP‐43 antibody (pS409/410, mouse monoclonal, 1:5000, CosmoBio USA, Carlsbad, CA, USA). For TDP‐43 pathology screening, we examined sections at the anterior commissure level, encompassing the amygdala, basal forebrain, putamen, globus pallidus, and hypothalamus. Small vessel disease was evaluated on H&E‐stained sections and diagnosed based on the presence of arteriosclerosis with microinfarcts, microhemorrhages, and ischemic white matter changes.^37^

**RESEARCH IN CONTEXT**

1. **Systematic review**: We conducted a PubMed search using the terms “dementia with Lewy bodies (DLB)” and “synuclein oligomers.” While several studies have investigated αSYN oligomers in rodent models or human cerebrospinal fluid, we found no studies that investigated the relationship between αSYN oligomers and VHs using autopsy brain tissue from patients with DLB.
2. **Interpretation**: Our findings demonstrate that patients with DLB who experienced VHs have more abundant αSYN oligomers in the parahippocampal cortex compared to those without VHs. These results provide evidence that the accumulation of αSYN oligomers in the parahippocampal cortex may be responsible for the development of VHs in DLB.
3. **Future directions**: Our findings warrant further validation in larger cohorts to determine whether αSYN oligomers in the parahippocampal cortex cause VHs in DLB. Our results suggest that αSYN oligomers could be therapeutic targets for VHs in DLB.

### 2.3 Brain regions of interest

To understand the underlying pathology of VH, we examined brain regions along the ventral visual stream.^38^ The analyzed regions included the primary visual cortex (V1, Brodmann area 17), visual association cortex (V2, Brodmann area 18), fusiform cortex, parahippocampal cortex, amygdala (including medial and lateral nuclei), and the nucleus basalis of Meynert (nbM). The amygdala and nbM were sampled at the level of the anterior commissure, while the parahippocampal cortex and fusiform cortex were sampled at the level of the lateral geniculate body.

### 2.4 Visualization of Lewy‐related pathology and αSYN oligomers

To detect Lewy‐related pathology, 5‐µm‐thick sections were processed with an autostainer (Thermo Lab Vision Autostainer 480S) using EnVision+ reagents (Dako, Carpinteria, CA, USA) and a phosphorylated αSYN antibody (1:10,000; mouse monoclonal, psyn#64, FUJIFILM Wako Pure Chemical Corporation, Osaka, Japan).

To visualize αSYN oligomers, αSYN‐PLA staining was employed as previously described with modifications.^22^, ^23^, ^39^, ^40^ We used Duolink kits (Sigma‐Aldrich, St. Louis, MO, USA) following the manufacturer's instructions. To generate two types of syn211‐PLA probes, αSYN antibody (syn211, mouse monoclonal, Abcam, Cambridge, UK) was conjugated with either of two oligonucleotides (plus or minus). After antigen retrieval using Target Retrieval Solution (pH 6, Agilent, Santa Clara, CA, USA) and blocking in Duolink blocking solution, sections were incubated with PLA probes (1:400) at 37°C for 1 h and then at 4°C overnight. Subsequent steps included ligation (37°C, 1 h), amplification (37°C, 2.5 h), and detection (room temperature, 1 h). After substrate development at room temperature for 20 min, the sections were counterstained with nuclear stain, dehydrated, and mounted.

### 2.5 Quantitative neuropathologic analysis

We performed quantitative neuropathologic analysis of Lewy‐related pathology and αSYN oligomers in brain regions along the ventral visual pathway. All stained slides were digitally scanned at 20× magnification using the Aperio ScanScope AT2 scanner (Leica Biosystems, Buffalo Grove, IL, USA). The scanned images were visualized and annotated using ImageScope (version 12.4.6; Leica Biosystems, Deer Park, IL, USA) and QuPath (version 0.5.0).^24^, ^41^ After delineating regions of interest for each brain region, we quantified phosphorylated αSYN using a color deconvolution algorithm to detect strongly immunoreactive DAB‐positive pixels. For αSYN‐PLA staining, we employed a custom positive pixel counting algorithm to quantify strongly stained NovaRed‐positive pixels. The analysis generated percentage burden values, representing the proportion of immunoreactive areas within the examined regions. All quantitative analyses were performed by investigators blinded to the patients' clinical status.

### 2.6 Genetic information

Genomic DNA was extracted from frozen cerebellar tissue using the AutoGen 245T platform. We determined *APOE* genotype based on two single‐nucleotide polymorphisms: rs7412 and rs429358.

### 2.7 Statistical analysis

Fisher exact tests were used to analyze categorical variables, whereas unpaired *t* tests were used for continuous variables. To examine interregional relationships, Spearman correlation analyses were performed to assess correlations of pathological burden across all examined brain regions. Statistical significance was defined as *p* < 0.05. Statistical analyses were performed using GraphPad Prism (version 10.1.0, GraphPad Software, La Jolla, CA, USA).

## 3 RESULTS

### 3.1 Patient characteristics

Demographic, clinical, and pathologic characteristics of the patients included in this study are summarized in Table 1. Patients with and without VHs showed no differences in age at onset of cognitive impairment, age at death, estimated duration of cognitive symptoms, and the interval between the final clinical evaluation and death. The *APOE* genotype distribution was identical between groups (ε2ε3: *n* = 1, each, ε3ε3: *n* = 3 each, ε3ε4: *n* = 1 each). Patients in the VH group developed VHs at a mean age of 77.7 ± 8.6 years, with VHs emerging, on average, 6.4 ± 5.3 years from the onset of cognitive impairment (2.4 ± 1.6 years before death). The interval between final neuropsychological assessment and death was 0.9 ± 0.4 years in the VH group and 1.4 ± 1.1 years in the non‐VH group (*p* = 0.34). The annual MMSE decline was −1.5 points in the non‐VH group and −0.64 points in the VH group, with no significant difference between groups (*p* = 0.54). Neuropathologic examination of the VH group confirmed three patients with diffuse Lewy body disease and two with limbic‐transitional Lewy body disease; the non‐VH group had two patients with diffuse Lewy body disease and three with limbic‐transitional Lewy body disease. Comorbid pathologies were similar across groups, including Braak neurofibrillary tangle stage, Thal amyloid phase, and presence of TDP‐43 pathology and small vessel disease.

**TABLE 1:** Demographic, clinical, and pathological characteristics of patients with dementia with Lewy bodies with and without visual hallucinations (VHs).

Features	VH(+) (*n* = 5)	VH(−) (*n* = 5)	*p* value
Males	5 (100%)	5 (100%)	–
Age at CI onset	71.2 ± 7.7	69.6 ± 4.5	0.68
Age at VH onset	77.7 ± 8.6	NA	–
Age at death	80.2 ± 7.2	78.6 ± 4.6	0.69
Interval from CI to death	8.9 ± 5.3	9.0 ± 3.7	0.97
Interval from CI to VH	6.4 ± 5.3	NA	–
Interval from VH to death	2.4 ± 1.6	NA	–
Interval from last visit to death	0.9 ± 0.4	1.4 ± 1.1	0.34
*APOE* ε4 carrier	1 (20%)	1 (20%)	> 0.99
Brain weight (g)	1280 ± 190	1190 ± 130	0.45
Diffuse/transitional LBD	3/2	2/3	> 0.99
Braak NFT stage	IV [III, V]	III [III, V]	0.81
Thal amyloid phase	3 [2, 5]	2 [1, 5]	0.95
TDP‐43 pathology	3 (60%)	1 (20%)	0.52
Small vessel disease	4 (80%)	3 (60%)	> 0.99

*Note*: Data are *n* (%), mean ± SD or median [25th and 75th percentile]; ages and intervals are presented in years.

Abbreviations: CI, cognitive impairment; LBD, Lewy body disease; NA, not applicable; NFT, neurofibrillary tangle; TDP‐43, transactive response DNA binding protein of 43 kDa; VH, visual hallucination.

### 3.2 αSYN oligomers

**FIGURE 1:**
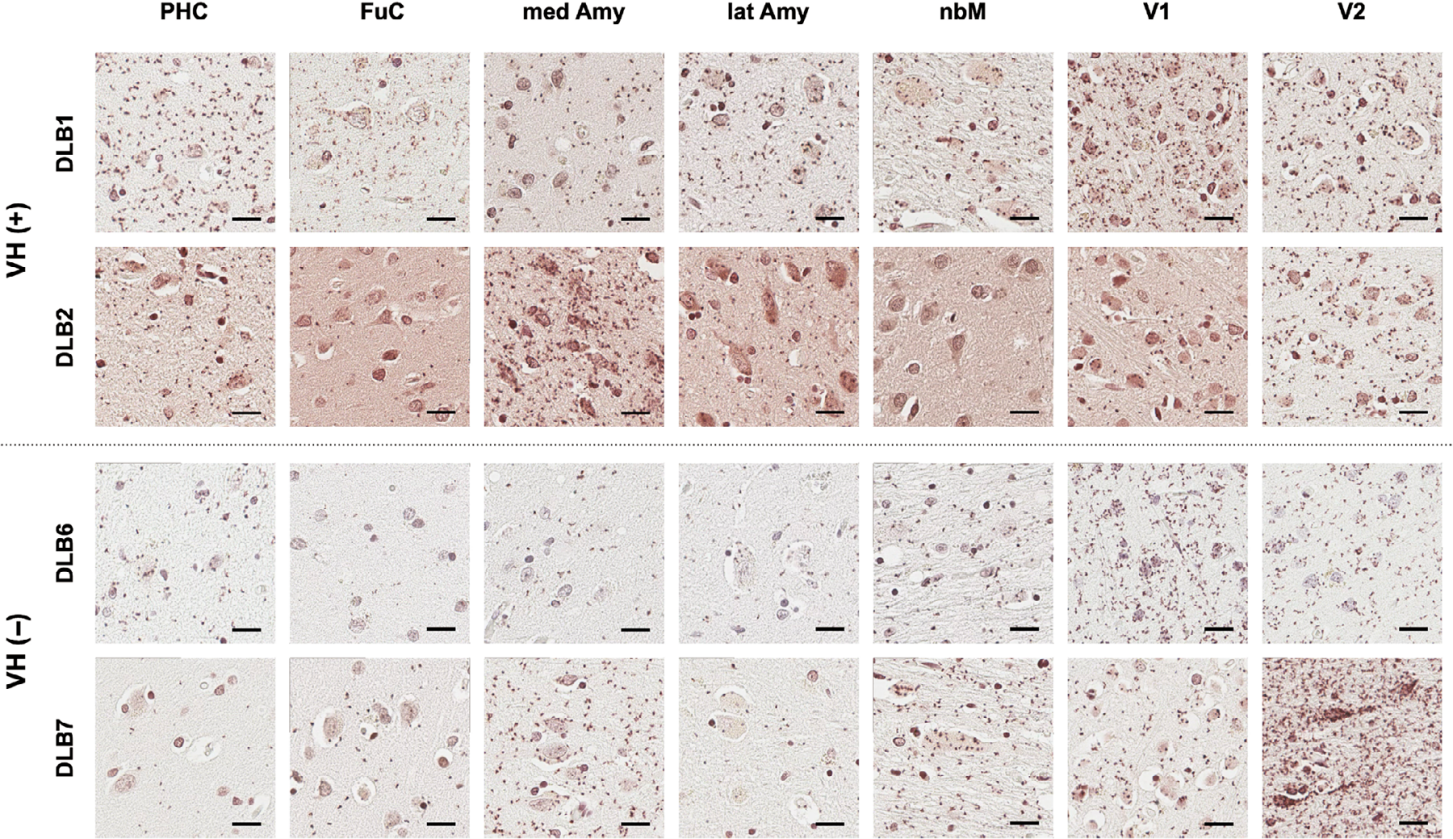
Representative images of αSYN‐PLA staining. Representative images showing αSYN oligomers in each brain region from patients with DLB with VHs (VH[+]; DLB1 and DLB2, or upper panels) and without VHs (VH[−]; DLB6 and DLB7, or lower panels). Red‐brown signals represent αSYN oligomers. αSYN oligomers are observed in both neuropil and neurons. Amy, amygdala; DLB, dementia with Lewy bodies; FuC, fusiform cortex; lat, lateral; med, medial; nbM, nucleus basalis of Meynert; PHC, parahippocampal cortex; PLA, proximity ligation assay; αSYN, α‐synuclein; V1, primary visual cortex; V2, secondary visual cortex; scale bar, 20 µm; VH, visual hallucination.

Figure 1 shows representative images of αSYN‐PLA staining in DLB patients with and without VHs. Red‐brown signals representing αSYN oligomers were detected in both neuropil and neurons. Abundant αSYN oligomers were identified in the parahippocampal cortex of patients with VHs but not in those without. The αSYN oligomer burden was similar across groups in the primary visual cortex and visual association cortex. Quantitative analyses of these findings are presented below.

### 3.3 Lewy‐related pathology

**FIGURE 2:**
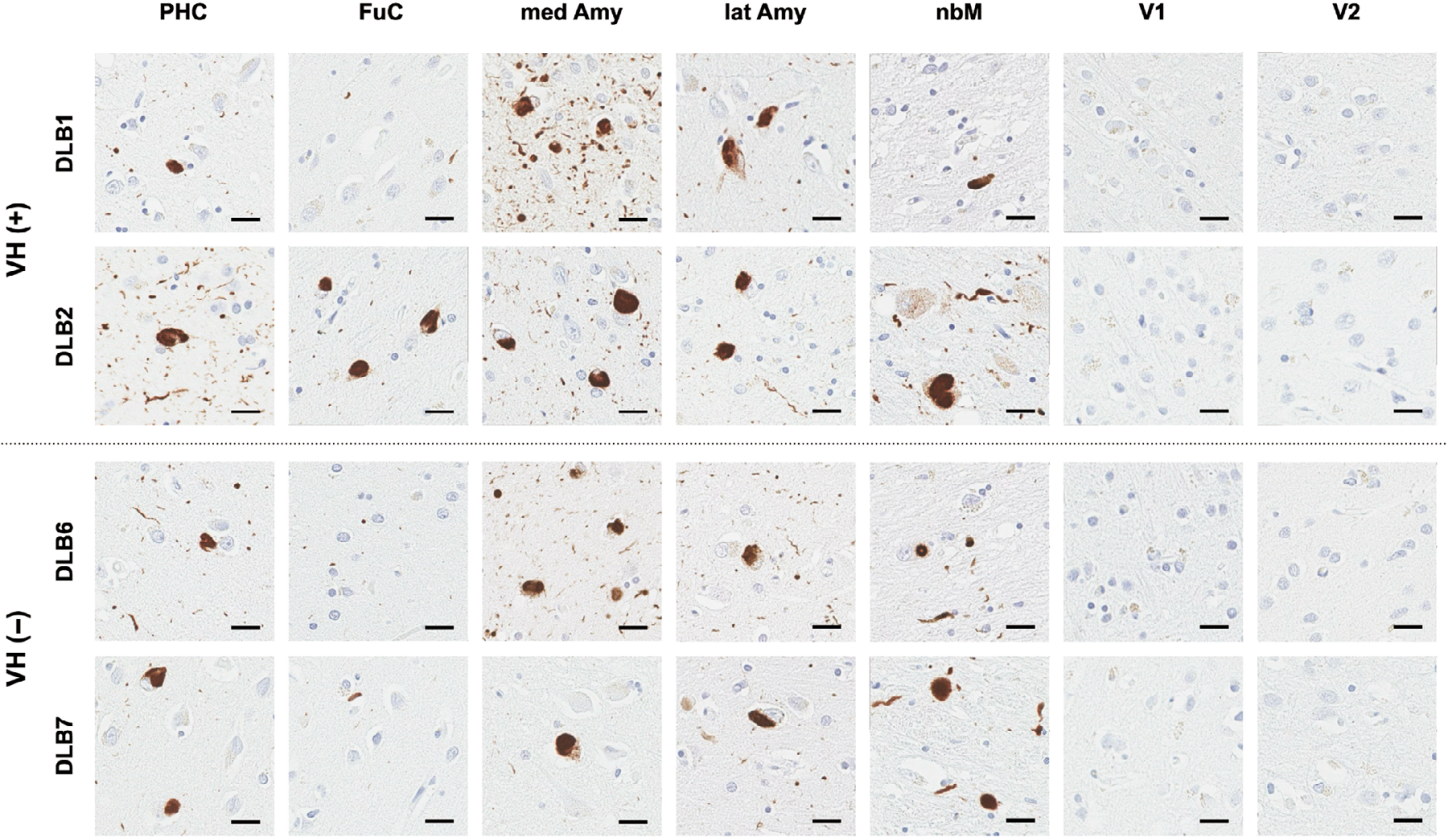
Representative images of phosphorylated αSYN immunohistochemistry. Representative images showing phosphorylated αSYN immunohistochemistry in each brain region from patients with DLB with visual hallucinations (VH) (VH[+]; DLB1 and DLB2, or upper panels) and without VH (VH[−]; DLB6 and DLB7, or lower panels). Lewy bodies and Lewy neurites are observed in the PHC, FuC, medial and lateral amygdala, and nbM. Lewy body‐related pathology is not found in the primary and secondary visual cortex. Amy, amygdala; DLB, dementia with Lewy bodies; FuC, fusiform cortex; lat, lateral; med, medial; nbM, nucleus basalis of Meynert; PHC, parahippocampal cortex; αSYN, α‐synuclein; V1, primary visual cortex; V2, secondary visual cortex; scale bar, 20 µm; VH, visual hallucination.

Figure 2 shows representative images of phosphorylated αSYN immunohistochemistry in patients with and without VHs. Lewy bodies and Lewy neurites showed a heavy burden in the subcortical and limbic regions of the parahippocampal cortex, amygdala, and nbM, with no group differences based on the presence or absence of VHs. Figure 2 demonstrates a small amount of Lewy‐related pathology in the fusiform cortex, as expected for those with only limbic‐transitional Lewy body disease, but also for those with diffuse distribution of Lewy‐related pathology. Additionally, no Lewy‐related pathology was observed in the primary visual cortex (V1, Brodmann area 17) or visual association cortex (V2, Brodmann area 18).

### 3.4 Comparison of pathology burden in DLB with and without VHs

**FIGURE 3:**
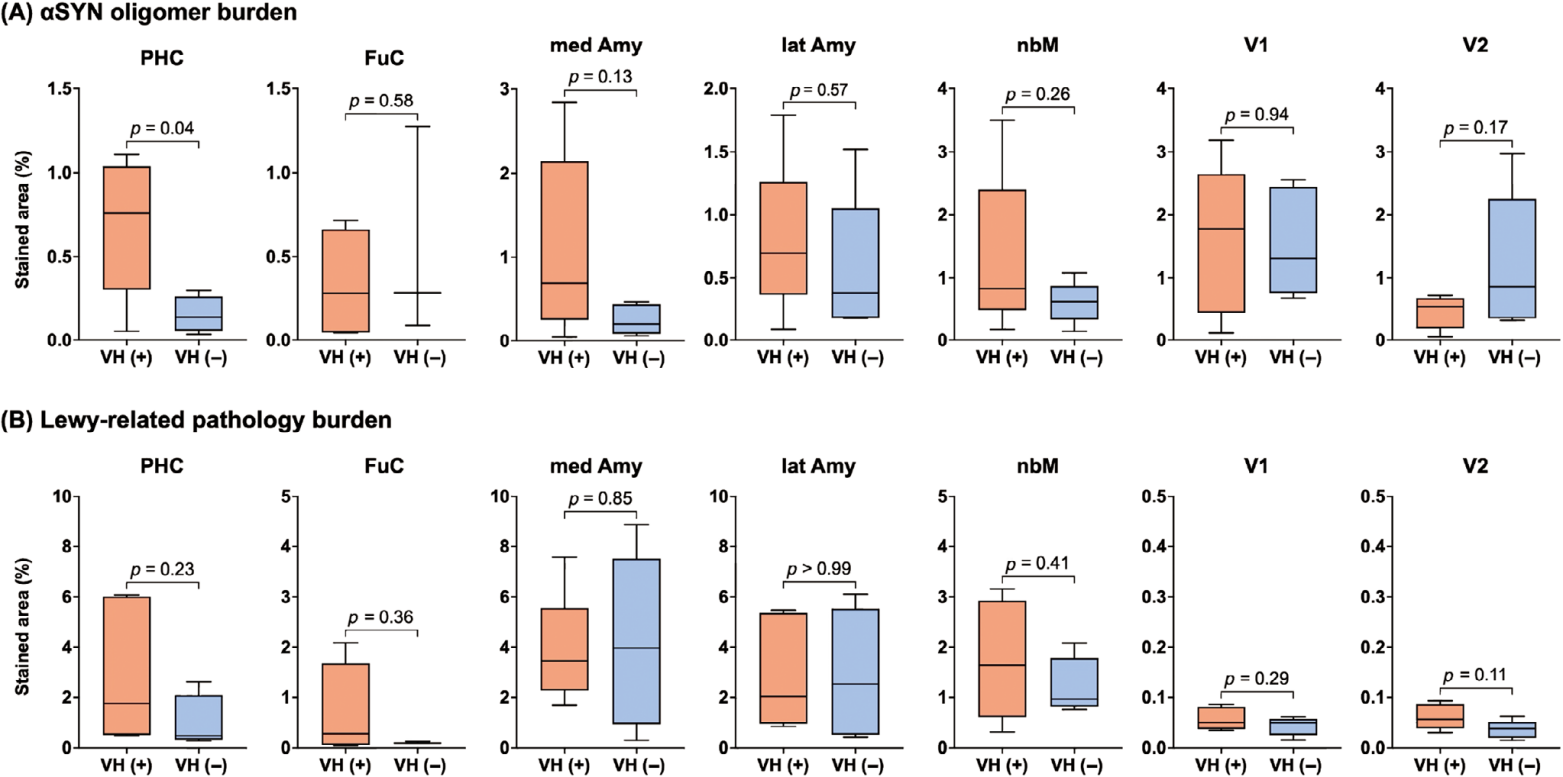
Comparison of stained areas. Quantitative neuropathologic analysis of (A) αSYN oligomer burden and (B) Lewy body‐related pathology burden in each brain region from patients with DLB with VHs (VH[+], red boxes) and without VHs (VH[−], blue boxes). The burden is indicated as the percentage of stained area. Only the PHC shows a significantly higher αSYN oligomer burden in patients with VHs compared to those without. No significant differences are observed in other brain regions for αSYN oligomer burden. In contrast, Lewy body‐related pathology burden is comparable across brain regions between patients with and without VHs. Box plots show median values with interquartile ranges and min‐max whiskers. Amy, amygdala; DLB, dementia with Lewy bodies; FuC, fusiform cortex; lat, lateral; med, medial; nbM, nucleus basalis of Meynert; PHC, parahippocampal cortex; αSYN, α‐synuclein; V1, primary visual cortex; V2, secondary visual cortex; VH, visual hallucinations.

To determine which brain regions and type of αSYN aggregates (αSYN oligomers or Lewy‐related pathology) were associated with VHs, we compared the percentage of pathologic burden of each pathology across brain regions for patients with and without VHs (Figure 3). DLB patients with VHs exhibited a greater burden of αSYN oligomers in the parahippocampal cortex compared to those without VHs (*p* = 0.041), while no significant differences were observed in other brain regions. To examine whether this finding was specific to VHs, we assessed correlations between parahippocampal αSYN oligomer burden and MMSE scores. There were no significant associations with annual MMSE decline (*r* = 0.15, *p* = 0.71) or last MMSE score (*r* = 0.085, *p* = 0.84). In contrast, the burden of Lewy‐related pathology was similar for DLB patients with and without VHs.

We examined interregional relationships of each pathological burden. Significant positive correlations were observed among regions for both αSYN oligomer burden (Figure S1A) and Lewy‐related pathology burden (Figure S1B). However, no significant correlations were observed between αSYN oligomer and Lewy‐related pathology burden across any regional pairs (Figure S1C).

## 4 DISCUSSION

In this study, we investigated whether the presence of VHs in DLB was associated with the regional burden of αSYN oligomers and Lewy‐related pathology. DLB patients with VHs had more abundant αSYN oligomers in the parahippocampal cortex than patients without VHs. Other regions comprising the ventral visual pathway showed no group differences, based on the presence or absence of VHs, in burden of αSYN oligomers. Regardless of VH status, the burden of Lewy‐related pathology was greater in limbic regions than in neocortical regions. Comparisons of limbic regions showed no difference in the burden of Lewy‐related pathology in the nbM, amygdala, or parahippocampal regions in patients with or without VHs during life.

Mechanisms that subserve VH include a sensory‐perceptual component associated with involvement of the limbic components of the ventral visual pathway.^42^ Direct electrical stimulation of the parahippocampal cortex has been shown to induce VHs of places and scenes.^43^ A functional magnetic resonance imaging (fMRI) study also provided evidence of the relationship between visual perceptual processing of images and scenes and its interface with memory systems.^44^ Another study using SPECT imaging in DLB showed greater complexity of VHs was associated with hypoperfusion of the anterior cingulate and parahippocampal regions.^45^ Furthermore, it has been reported that patients with locked‐in syndrome who experienced hallucinations showed selective cortical volume reduction in the parahippocampal gyrus.^46^ These studies suggest that the parahippocampal region plays a significant role in the occurrence of VHs, through both functional and structural mechanisms. In this study, Lewy‐related pathology burden in the parahippocampal cortex was a common feature of DLB; however, the burden of Lewy‐related pathology did not differentiate patients with and without VHs in our study. In contrast, αSYN oligomers showed greater burden in the parahippocampal cortex of patients with VHs. This suggests that αSYN oligomers may represent an earlier and more specific pathological marker for VH development in DLB, potentially preceding the formation of mature Lewy bodies. When examined in vivo, αSYN oligomers are more toxic to neurons.^16^ Fusco et al. identified structural features enabling αSYN oligomers to disrupt biological membranes and cellular functions, and this highlights the lipophilic elements that interact with lipid bilayers.^18^ Cascella et al. showed that αSYN fibrils released toxic oligomeric species that made neuronal membranes more permeable, causing cellular damage and dysfunction.^20^ The accumulation of these toxic oligomers in the parahippocampal cortex may interfere with the normal processing of visual information and memory integration, potentially leading to the manifestation of VHs in patients with DLB.

Although no previous studies examined the relationship between αSYN oligomers and VH, several investigations have explored the correlation between Lewy‐related pathology and VHs. Harding et al. counted Lewy bodies detected by anti‐αSYN and anti‐ubiquitin immunohistochemistry and reported significantly higher Lewy body counts in the parahippocampal gyrus and amygdala in PDD/DLB patients with VHs compared to those without VHs.^11^ Of note, they also showed that when stratifying patients based on VH onset, patients with late‐onset VHs showed no significant differences in Lewy body counts across any brain regions compared to those without VHs.^11^ Our DLB cohort included patients with a relatively short interval between VH onset and death (mean 2.4 years), which likely corresponds to late‐onset VHs in the study by Harding et al. Consistent with their findings, we observed no significant differences in Lewy‐related pathology between patients with and without VHs. However, we did observe a significant increase in αSYN oligomers in the parahippocampal cortex in patients with VHs. This apparent discrepancy can be explained by the temporal transition of αSYN pathology that we previously characterized.^23^, ^40^ In PD, brain regions affected early in the disease course had reduced αSYN oligomers but abundant Lewy‐related pathology, while regions affected later had abundant oligomers but less Lewy‐related pathology.^23^ Furthermore, we demonstrated an inverse correlation between αSYN oligomer and Lewy‐related pathology burdens in LRRK2‐related PD.^40^ Applying this framework to the present study, the relatively short interval between VH onset and death in our cohort likely provided a critical window to detect αSYN oligomer accumulation. This temporal transition should be taken into consideration when examining correlations between specific symptoms and αSYN pathology. Specifically, toxic αSYN oligomers initially accumulate, causing neuronal dysfunction and developing symptoms corresponding to their regional accumulation.^23^ Over time, however, αSYN aggregates transition from αSYN oligomers to Lewy‐related pathology, potentially making Lewy‐related pathology more prominent when analyzing autopsy brains examined long after symptom onset.

A recent study using principal component analysis identified an occipital–superior temporal pattern of conventional αSYN pathology (KM51 antibody) associated with the hallucination subdomain score of the Neuropsychiatric Inventory, while parahippocampal pathology loaded on a separate component not associated with that.^47^ These divergent regional associations may reflect differences in the pathological entities examined (mature Lewy‐related pathology vs early‐stage oligomers) and underscore the importance of considering disease stage when examining pathology–symptom relationships.

A primary limitation of this study is the small sample size, which may have led to reduced power to detect differences with smaller effect sizes. Our cohort consisted exclusively of male patients, which limits the generalizability of our findings. Additionally, we conducted multiple statistical comparisons in several brain regions without correction for multiple testing. While our analyses were hypothesis‐driven and focused on functionally distinct regions within the ventral visual pathway, we acknowledge the potential for type I error. Further investigations with larger sample sizes that include both sexes are warranted to further inform the relationship between αSYN oligomers and VHs. Moreover, as an observational clinicopathological study, we did not investigate the detailed mechanisms by which αSYN oligomer accumulation leads to VHs. Future studies are warranted to address this question.

## 5 CONCLUSION

In conclusion, this study demonstrates that the accumulation of αSYN oligomers in the parahippocampal cortex may contribute to the development of VHs in DLB. This observation provides additional evidence linking αSYN oligomers to one of the core clinical symptoms in DLB using *post mortem* human brain samples. Future studies are warranted to investigate whether therapeutic strategies targeting αSYN oligomers may improve VHs in patients with DLB.

## CONFLICT OF INTEREST STATEMENT

Hiroaki Sekiya is funded through fellowships from the Japanese Society of Neurology, the Cell Science Research Foundation, and the Uehara Memorial Foundation. He is partially supported by the American Parkinson Disease Association (APDA) Research Grant, the Multiple System Atrophy Coalition Research Grant, the State of Florida Ed and Ethel Moore Alzheimer's Disease Research Program (24A08), and Jaye F. and Betty F. Dyer Foundation Fellowship in progressive supranuclear palsy research. Lukas Franke, Daisuke Ono, and Michael DeTure report no conflicts of interest. Owen A. Ross receives research support from the National Institutes of Health (NIH), the APDA, the Department of Defense, and the Little Family Foundation and serves as a consultant for SciNeuro Consulting. Gregory S. Day receives research support from the NIH, serves as a topic editor on dementia for DynaMed Plus (EBSCO Industries, Inc.), is the clinical director for the Anti‐NMDA Receptor Encephalitis Foundation (uncompensated), has provided record review and expert medical testimony on legal cases pertaining to the management of Wernicke encephalopathy, and holds stocks (worth over $10,000) in ANI Pharmaceuticals (a generic pharmaceutical company). Christian Lachner receives research support from the NIH. Neill R. Graff‐Radford receives research support from NIH, Lilly, Biogen, Novartis, and Abbvie; Pamela J. McLean receives research support from NIH. Tanis J. Ferman receives research support from NIH and serves as a consultant for Acadia pharmaceuticals. Dennis W. Dickson receives research support from NIH. Author disclosures are available in the supporting information.

## CONSENT STATEMENT

Brain autopsies were performed with the consent of the legal next of kin or an individual with legal authority to grant permission for autopsy. De‐identified studies using autopsy samples are considered exempt from human subject research by the Mayo Clinic Institutional Review Board.

## Supporting information



Supporting Information

Supporting Information
