# Supplementary material for: Association between visual hallucinations and α‐synuclein oligomers in patients with dementia with Lewy bodies
Source: Alzheimers Dement. 2025 Nov 14;21(11):e70904. doi: 10.1002/alz.70904 (PMC12616884; doi:10.1002/alz.70904)
Supplement: Supplementary file 2 — Supporting Information [file ALZ-21-e70904-s002.pdf]

Supplementary material

Association between Visual Hallucinations and α-Synuclein Oligomers in Patients with Dementia with Lewy Bodies

Hiroaki Sekiya, M.D., Ph.D.[1], Lukas Franke, M.D.[1], Daisuke Ono, M.D., Ph.D.[1], Michael DeTure, Ph.D.[1], Owen A. Ross, Ph,D.[1], Gregory S. Day, M.D.[2], Christian Lachner, M.D.[2,3], Neill R Graff-Radford, M.D.[2], Pamela J. McLean, Ph.D.[1], Tanis J. Ferman, Ph.D.[3], Dennis W. Dickson, M.D.[1]

[1] Department of Neuroscience, Mayo Clinic, 4500 San Pablo Rd, Jacksonville, Florida 32224, USA
[2] Department of Neurology, Mayo Clinic, 4500 San Pablo Rd, Jacksonville, Florida 32224, USA
[3] Department of Psychiatry & Psychology, Mayo Clinic, 4500 San Pablo Rd, Jacksonville, Florida 32224, USA

*Corresponding author: Hiroaki Sekiya, M.D., Ph.D.

Supplementary Figure 1

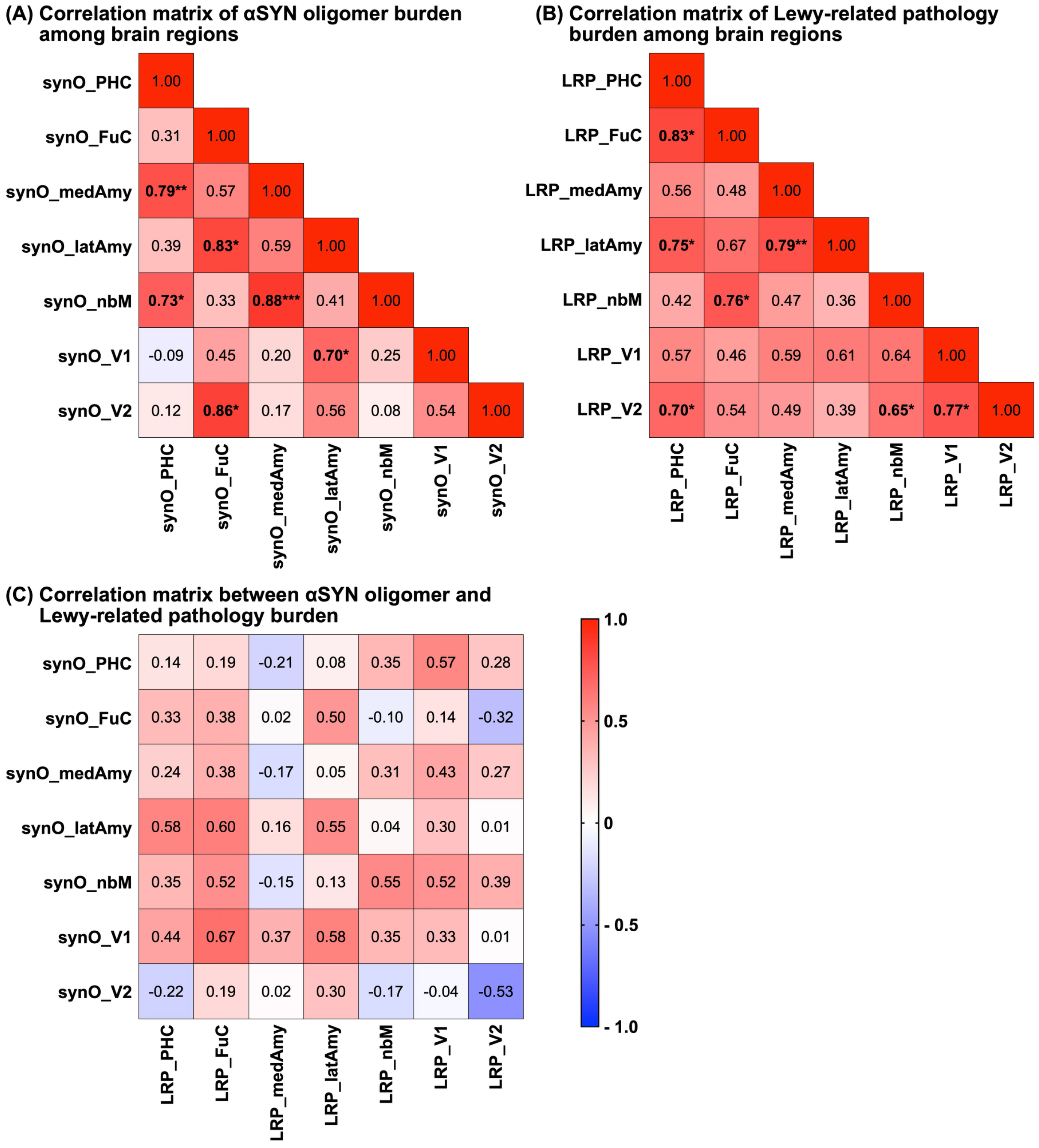

Heatmaps showing Spearman correlation coefficients (r) for (A) αSYN oligomer burden among regions, (B) Lewy-related pathology burden among regions, and (C) between αSYN oligomer and Lewy-related pathology burden. Significant positive correlations (* $p < 0.05$; ** $p < 0.01$. *** $p < 0.001$) are observed within each pathological category, while no significant correlations are observed between the two pathological categories. Color scale ranges from r = -1.0 (blue) to r = 1.0 (red). *αSYN*, α-synuclein; *synO*, α-synuclein oligomer; *LRP*, Lewy-related pathology; *PHC*, parahippocampal cortex; *FuC*, fusiform cortex; *medAmy*, medial amygdala; *latAmy*, lateral amygdala; *nbM*, nucleus basalis of Meynert; *V1*, primary visual cortex; *V2*, secondary visual cortex.
